# *In Vivo* Metabolism Study of Xiamenmycin A in Mouse Plasma by UPLC-QTOF-MS and LC-MS/MS

**DOI:** 10.3390/md13020727

**Published:** 2015-01-28

**Authors:** Feng Lei, Du Gao, Xi Zhang, Jun Xu, Min-Juan Xu

**Affiliations:** 1 Key Laboratory of Systems Biomedicine, Shanghai Center for Systems Biomedicine, Shanghai Jiao Tong University, Shanghai 200240, China; E-Mail: fiona.fenglei@sjtu.edu.cn; 2 Instrumental Analysis Center, Shanghai Jiao Tong University, Shanghai 200240, China; 3 State Key Laboratory of Microbial Metabolism and School of Life Science & Biotechnology, Shanghai Jiao Tong University, Shanghai 200240, China; E-Mails: huanranfanfei@163.com (D.G.); xujunn@sjtu.edu.cn (J.X.); 4 Waters Corporation, Building 13, No. 1000 Jinhai Road, Pudong New District, Shanghai 201206, China; E-Mail: xi_zhang@waters.com; 5 Institute of Oceanology, Shanghai Jiao Tong University, Shanghai 200240, China

**Keywords:** *Streptomyces xiamenensis*, xiamenmycin, benzopyran, antifibrosis

## Abstract

Xiamenmycin A is an antifibrotic leading compound with a benzopyran skeleton that is isolated from mangrove-derived *Streptomyces xiamenensis.* As a promising small molecule for fibrotic diseases, less information is known about its metabolic characteristics *in vivo*. In this study, the time-course of xiamenmycin A in mouse plasma was investigated by relative quantification. After two types of administration of xiamenmycin A at a single dose of 10 mg/kg, the plasma concentrations were measured quantitatively by LC-MS/MS. The dynamic changes in the xiamenmycin A concentration showed rapid absorption and quick elimination in plasma post-administration. Four metabolites (M1–M4) were identified in blood by UPLC-QTOF-MS, and xiamenmycin B (M3) is the principal metabolite *in vivo*, as verified by comparison of the authentic standard sample. The structures of other metabolites were identified based on the characteristics of their MS and MS/MS data. The newly identified metabolites are useful for understanding the metabolism of xiamenmycin A *in vivo*, aiming at the development of an anti-fibrotic drug candidate for the therapeutic treatment of excessive fibrotic diseases.

## 1. Introduction

**Figure 1:**
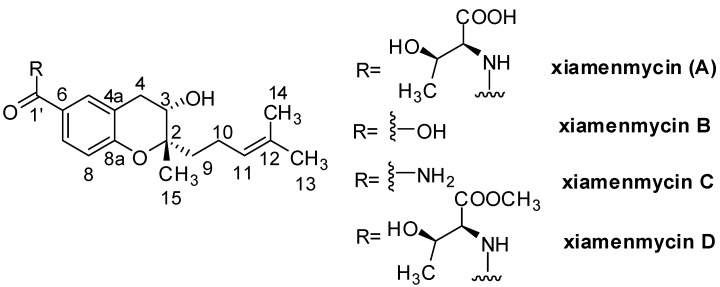
Chemical structures of xiamenmycins A–D.

Fibrosis, as a result of chronic inflammatory reactions induced by various stimuli, has gained increasing attention [1]. However, successful methods for treating fibrosis have been limited, and a lack of effective small-molecule medicines is one of the serious problems [2,3]. Therefore, searching for a bioactive leading compound from natural resources represents an emerging pharmacological and therapeutic area for excessive fibrotic diseases. The treasure trove of natural products produced by mangrove-derived actinomycetes is of great interest to drug developers. There is unambiguous evidence that a large diversity of structurally unique bioactive compounds have been obtained from mangrove-derived actinomycetes [4,5,6,7]. In our work, a series of benzopyran derivatives, named xiamenmycin A–D, were discovered from mangrove-derived *Streptomyces xiamenensis* as potential drug candidates against excessive fibrotic diseases (Figure 1) [8,9,10,11]. Xiamenmycin A, also named xiamenmycin, significantly attenuated hypertrophic scar formation and had no significant toxic effect on mice using a mechanical stretch-induced mouse hypertrophic scar model [10]. For the mechanism of fibrogenesis, it is well known that self-perpetuating circuits of inflammation and ECM accumulation and constriction by inflammation and mechanical force directly influence the development of fibrotic diseases [4]. Xiamenmycin A could suppress local inflammation by reducing CD4^+^ lymphocyte and monocyte/macrophage retention in fibrotic foci and block fibroblast adhesion with monocytes [10]. Hence, the leading compounds aimed at these circuits may have targeting therapeutic effects on fibrotic diseases, while avoiding severely disturbing physiological processes induced by steroidal anti-inflammatory drugs.

The promising anti-fibrotic activities of xiamenmycins have triggered our efforts to determine their structure, increase their structural diversity, identify the gene cluster responsible for their biosynthesis and improve their production by ribosome engineering. The absolute configurations of xiamenmycins were determined by extensive spectroscopic data analyses, Mosher’s method, Marfey’s reagent and quantum mechanical calculations [8]. The biosynthetic pathway was elucidated, and all of the genes in the pathway were genetically and biochemically characterized [12]. Spontaneous rifampicin resistance combined with streptomycin resistance by introducing a mutated *rpsL* gene into *S. xiamenensis* was performed to increase the production of secondary metabolites [9]. However, as a promising leading compound, scant information is known concerning the pharmacokinetic and metabolic characteristics of xiamenmycins.

Our study is focused on the investigation of the metabolism of xiamenmycin A, including the mass fragment analysis of a series of xiamenmycins, dynamic changes in the xiamenmycin A concentration in plasma after intraperitoneal (i.p.) and intravenous (i.v.) administration, as well as the metabolites *in vivo*. The study may provide the chemical data to identify xiamenmycins rapidly in plasma, preliminary pharmacokinetic information of xiamenmycin A for future pharmacology studies and the structures of metabolites *in vivo* to further understand the metabolism of xiamenmycin A as a drug candidate.

## 2. Results

### 2.1. Fragmentation Patterns of Xiamenmycins

The chemical structure of xiamenmycin A is composed of three moieties: threonine-substituted groups, a prenylated side chain and a benzopyran skeleton. Both the negative and positive modes of ESI mass spectra of xiamenmycins were examined in this study. In principle, fewer product ions were observed in the negative mode, while [M + H]^+^ ions of sufficient abundance can be used in MS/MS experiments and provide more structural information. The fragmentation pattern analyses of xiamenmycins A–D are important for the metabolite characterization. Accurate mass measurements of pseudo-molecular ions are listed in Table 1.

**Table 1:** Accurate mass measurement for xiamenmycins in positive and negative modes.

No.	Formula	Retention Time (min)	Positive *m*/*z* Calculated	Positive *m*/*z* Found	Error (ppm)	Negative *m*/*z* Calculated	Negative *m*/*z* Found	Error (ppm)
**Xiamenmycin A**	C_21_H_29_NO_6_	4.24	392.2073	392.2072	−0.3	390.1917	390.1915	-0.5
**Xiamenmycin B**	C_17_H_22_O_4_	4.93	291.1596	291.1580	−5.5	289.1440	289.1441	0.3
**Xiamenmycin C**	C_17_H_23_NO_3_	4.31	290.1756	290.1738	−6.2	334.1659 *	334.1654 *	1.5
**Xiamenmycin D**	C_22_H_31_NO_6_	4.67	406.2230	406.2194	−8.9	404.2073	404.2082	2.2

* [M + HCOO^−^]^−^ ion in the negative scan mode. The molecular formula is C_18_H_24_NO_5_.

**Figure 2:**
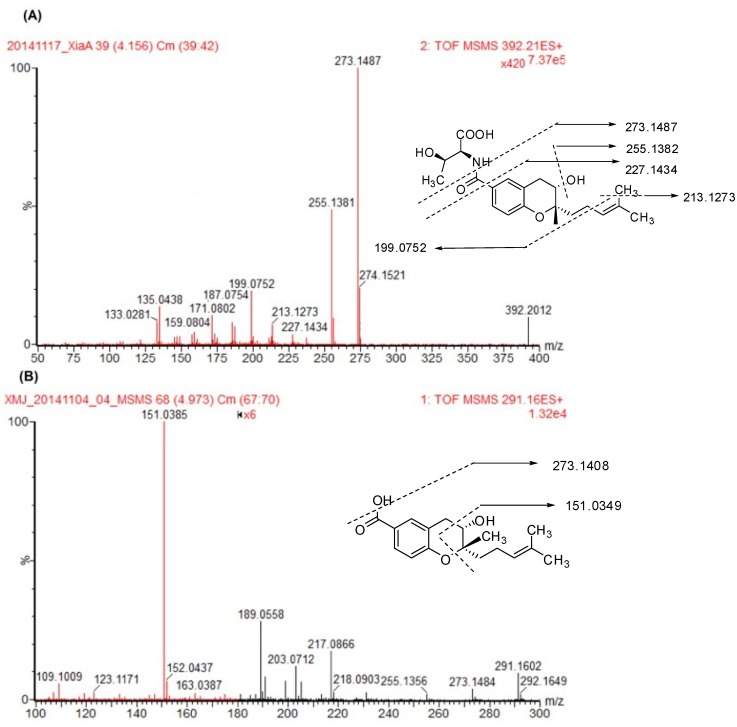
Secondary mass spectra of xiamenmycins A (**A**) and B (**B**).

A full-scan mass spectrum of xiamenmycin A under low collision energy showed the pseudo-molecular ion [M + H]^+^ at *m*/*z* 392.2072 in the positive mode. Under high-collision energy, the daughter ions of xiamenmycin A at *m*/*z* 273.1487, 255.1382, 227.1434, 213.1273, 199.0752, 187.0754, 171.0802, 159.0804, 135.0438 and 133.0281 were shown (Figure 2A). The highest abundant fragment ion at *m*/*z* 273.1487 was formed by the deletion of an amino acid moiety (-C_4_H_9_NO_3_, *m*/*z* 119), which was also observed in the low-collision mass spectrum. The molecular ion at *m*/*z* 255.1382 was produced by the sequential loss of H_2_O, possibly at C3, with the formation of a double bond. The further fragment ions at *m*/*z* 227.1434, 213.1273 and 199.0752 were formed by the loss of the carbonyl group at C6, the methyl group and the loss of 2-methylprop-1-ene from the prenylated side chain, respectively. The fragment ion of *m*/*z* 135.0438 presented the benzopyran skeleton. Xiamenmycin D is the methyl ester of xiamenmycin A, with the pseudo-molecular ion [M + H]^+^ at *m*/*z* 406.2194, and both compounds shared similar mass fragments at *m*/*z* 227.1476, 255.1377, 227.1431, 213.1262, 199.0757, 187.0763, 171.0811, 159.0810, 135.0448 and 133.0290, as well as similar relative abundance ratios (Table 2 and Supplementary Figure S5). Therefore, xiamenmycin A and D have the same fragmentation pattern.

Xiamenmycin B showed the pseudo-molecular ion [M + H]^+^ at *m*/*z* 291.1580 in the positive mode. In the secondary mass spectrum of xiamenmycin B, its daughter ions were at *m*/*z* 273.1484, 255.1356, 217.0866, 203.0712, 189.0558 and 151.0385 (Figure 2B). The highest abundant fragment of xiamenmycin B was at *m*/*z* 151.0385, formed by direct cleavage of the pyran ring. The MS/MS fragment at *m*/*z* 273.1484 and 217.0866 was also found, corresponding to the loss of H_2_O and sequential loss of the -C_4_H_8_ unit from the isoprenoid side chain. Another MS/MS fragment with low abundance at *m*/*z* 255.1356 was also the sequential loss of H_2_O from 273.1484, which is also observed in the mass spectrum of xiamenmycin A, but with high relative abundance. Xiamenmycin C is different from xiamenmycin B at the substituent group of the benzoic ring at position 4, with the pseudo-molecular ion [M + H]^+^ at *m*/*z* 290.1738. The hydroxy group was replaced by the amino group. Due to the proposed fragmentation process, a major fragment ion at *m*/*z* 150.0547 was observed in the MS/MS spectrum of xiamenmycin C, which is in accordance with the different substituent group, *i.e.*, from -OH to -NH_2_ at C1′ (Table 2 and Supplementary Figure S4). Xiamenmycin B and C have the same pyran ring cleavage fragmentation pattern, which is different from xiamenmycin A and D.

**Table 2:** Fragment ions of xiamenmycins A–D in the positive mode.

	*m*/*z* Found	*m*/*z* Calculated	% Base	Formula	mDa	ppm
xiamenmycin A	273.1487	273.1491	100	C_17_H_21_O_3_	−0.4	−1.5
	255.1382	255.1385	50	C_17_H_19_O_2_	−0.4	−1.6
	227.1434	227.1436	4	C_16_H_19_O	−0.2	−0.9
	213.1273	213.1279	8	C_15_H_17_O	−0.6	−2.8
	199.0752	199.0759	20	C_13_H_11_O_2_	−0.7	−3.5
	187.0754	187.0759	9	C_12_H_11_O_2_	−0.5	−2.7
	171.0802	171.0810	13	C_12_H_11_O	−0.8	−4.7
	159.0804	159.0810	5	C_11_H_11_O	−0.6	−3.8
	135.0438	135.0446	15	C_8_H_7_O_2_	−0.8	−5.9
	133.0281	133.0290	10	C_8_H_5_O_2_	−0.9	−6.8
xiamenmycin B	273.1484	273.1491	1	C_17_H_21_O_3_	−0.7	−2.6
	255.1356	255.1385	0.5	C_17_H_19_O_2_	−2.9	−11.4
	217.0866	217.0865	3	C_13_H_13_O_3_	0.1	0.5
	203.0712	203.0708	2	C_12_H_11_O_3_	0.4	2
	189.0558	189.0552	5	C_11_H_9_O_3_	0.6	3.2
	151.0385	151.0395	100	C_8_H_7_O_3_	−1	−6.6
xiamenmycin C	273.1488	273.1491	4	C_17_H_21_O_3_	−0.3	−1.1
	255.1381	255.1385	4	C_17_H_19_O_2_	−0.4	−1.6
	216.1061	216.1025	3	C_13_H_14_NO_2_	3.2	14.8
	204.1025	204.1025	5	C_12_H_14_NO_2_	0.2	1
	188.0730	188.0712	3	C_11_H_10_NO_2_	1.8	9.6
	173.0960	173.0966	5	C_12_H_13_O	−0.6	−3.5
	150.0547	150.0555	100	C_8_H_8_NO_2_	−0.8	−5.3
xiamenmycin D	273.1476	273.1491	100	C_17_H_21_O_3_	−1.5	−5.5
	255.1377	255.1385	48	C_17_H_19_O_2_	−0.8	−3.1
	227.1431	227.1436	4	C_16_H_19_O	−0.5	−2.2
	213.1262	213.1279	7	C_15_H_17_O	−1.7	−8
	199.0757	199.0759	16	C_13_H_11_O_2_	−0.2	−1
	187.0763	187.0759	7	C_12_H_11_O_2_	0.4	2.1
	171.0811	171.0810	9	C_12_H_11_O	0.1	0.6
	159.0810	159.0810	3	C_11_H_11_O	0	0
	135.0448	135.0446	12	C_8_H_7_O_2_	0.2	1.5
	133.0290	133.0290	6	C_8_H_5_O_2_	0	0

### 2.2. Dynamic Changes of Xiamenmycin A in Plasma Post-Administration

According to our previous investigation, xiamenmycin A was intraperitoneally injected into animals; thus, the i.p. approach seems to be one of the possible methods of administration [10]. Here, the relative quantification of its concentrations in plasma was measured to compare the differences between i.v. and i.p. administration. The linearity of the detector response was examined by analyzing a series of xiamenmycin A solutions at ten different concentrations. Calibration curves for xiamenmycin A in the plasma matrix were shown to have a linear regression in the concentration range from 1 to 5000 ng/mL. The coefficient of determination (*r*^2^) was 0.9956, which indicated good linear regression in the concentration range. The lower limit of quantification was 1 ng/mL for xiamenmycin A.

**Figure 3:**
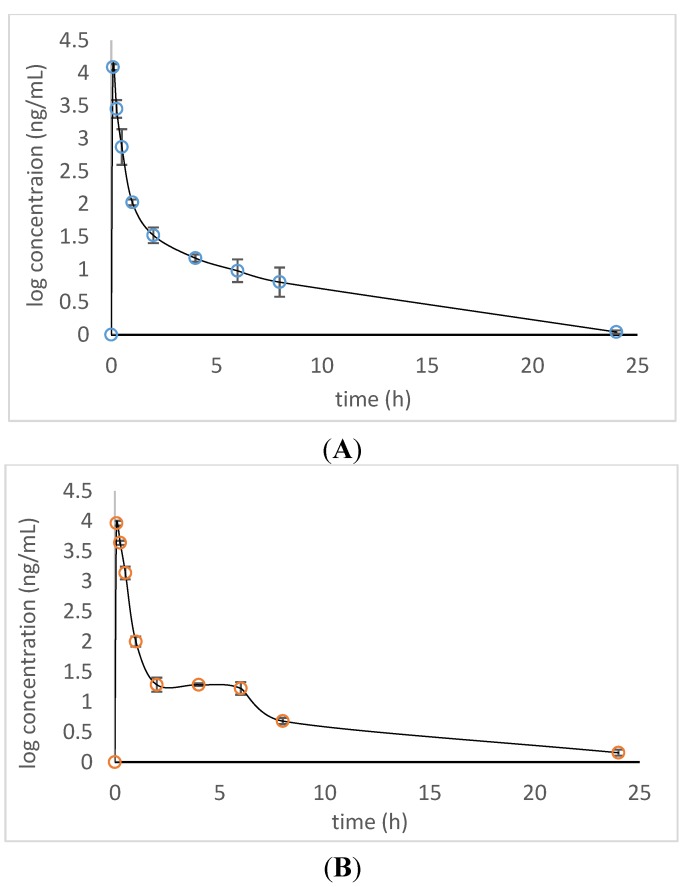
Plot of the observed xiamenmycin A concentration *versus* time of post-administrations (*n* = 3): i.v. (**A**); i.p. (**B**).

A time course of the absorbed xiamenmycin A in plasma was primarily evaluated for future pharmacokinetic experiments (Figure 3). It was found that xiamenmycin A can be absorbed into blood rapidly in plasma after i.p. administration and reached a maximal concentration in blood after 5 min. There was a difference between the two types of administrations, *i.e.*, the blood concentration of xiamenmycin A remained at 10–30 ng/mL for four hours after i.p. administration. However, xiamenmycin A was still eliminated quickly and decreased to *ca*. 100 ng/mL at 1 h post-administration. The investigations of dynamic changes of xiamenmycin A in plasma post-administration indicated that formulation selection is crucial for the improvement of its biological half-life in blood.

### 2.3. Xiamenmycin A-Related Metabolites Detected in Mouse Plasma

**Figure 4:**
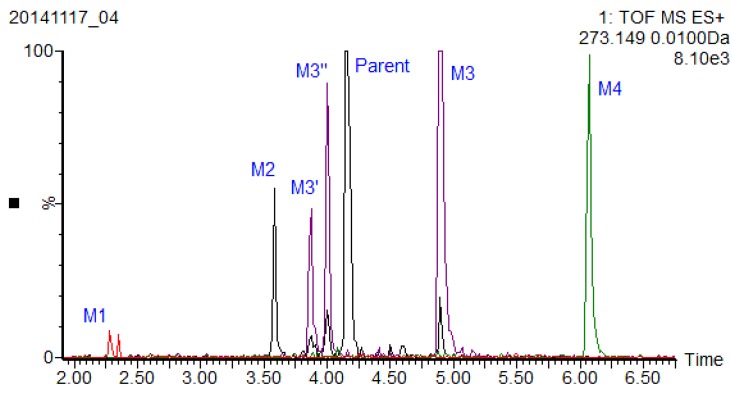
Extracted ion chromatography of metabolites related to xiamenmycin A in the overlay mode.

UPLC-QTOF-MS and UPLC-QTOF-MS/MS are broadly applicable for metabolite identification in drug development [13,14,15]. A rapid and sensitive UPLC-QTOF-MS method was developed to detect xiamenmycin A-related metabolites in plasma. The extracted ion chromatography of metabolites with a benzopyran skeleton is shown in the overlay mode (Figure 4). As a result, M3 was identified as xiamenmycin B by comparison of the retention time and fragment ions with an authentic standard sample, and the other three metabolites in mouse plasma derived from xiamenmycin A were tentatively identified by accurate mass and fragment ions, as listed in Table 3.

**Table 3:** Accurate mass measurement for pseudo-molecular and fragment ions of xiamenmycin A’s metabolites in plasma.

	Pseudo-Molecular Ion (PI)						Fragment Ion (MS/MS)					
	Rt (min)	*m*/*z* Found	*m*/*z* Calculated	Formula	mDa	ppm	*m*/*z* Found	*m*/*z* Calculated	% Base	Formula	mDa	ppm
M1	2.28	426.2125	426.2128	C_21_H_32_NO_8_	−0.3	−0.7	307.1574	307.1572	100	C_20_H_21_NO_2_	0.2	0.7
							289.1471	289.1467	30	C_20_H_19_NO	0.4	1.4
							271.1334	271.1334	85	C_17_H_19_O_3_	0	0
M2	3.58	273.1493	273.1491	C_17_H_21_O_3_	0.2	0.7	255.1406	255.1385	28	C_17_H_19_O_2_	2.1	8.2
							199.0749	199.0759	28	C_13_H_11_O_2_	−1	−5
							171.083	171.0810	35	C_12_H_11_O	2	11.7
							147.0454	147.0446	20	C_9_H_7_O_2_	0.8	5.4
							135.0437	135.0446	50	C_8_H_7_O_2_	−0.9	−6.7
							133.0275	133.0290	100	C_8_H_5_O_2_	−1.5	−11.3
M3	4.89	291.1588	291.1596	C_17_H_23_O_4_	−0.8	−2.7	273.1506	273.1491	2	C_17_H_21_O_3_	1.5	5.5
							255.1398	255.1385	0.5	C_17_H_19_O_2_	1.3	5.1
							217.0863	217.0865	4	C_13_H_13_O_3_	−0.2	−0.9
							203.0726	203.0708	2	C_12_H_11_O_3_	1.8	8.9
							189.0559	189.0552	6	C_11_H_9_O_3_	0.7	3.7
							151.0389	151.0395	100	C_8_H_7_O_3_	−0.6	−4
M4	6.07	305.1755	305.1753	C_18_H_25_O_4_	0.2	0.7	273.1480	273.1491	7	C_17_H_21_O_3_	−1.1	−4
							255.1382	255.1385	1	C_17_H_19_O_2_	−0.3	−1.2
							217.0881	217.0865	5	C_13_H_13_O_3_	1.6	7.4
							203.0718	203.0708	9	C_12_H_11_O_3_	1	4.9
							189.0524	189.0552	1	C_11_H_9_O_3_	−2.8	−14.8
							165.0544	165.0552	100	C_9_H_9_O_3_	−0.8	−4.8

M1, eluted at 2.28 min, showed the predominant quasi-molecular ion [M + H]^+^ at *m/z* 426.2128 (C_21_H_32_NO_8_) that is formed by oxidized alkenes to dihydrodiol. The major fragment at *m/z* 307.1572 (C_20_H_21_NO_2_) was produced by further loss of the threonine moiety, and 271.1334 (C_17_H_19_O_3_) was produced by the sequential loss of two oxygens, possibly from the oxidized prenylated side chain.

M2, eluted at 3.58 min, showed the predominant quasi-molecular ion [M + H]^+^ at *m*/*z* 273.1493 (C_17_H_21_O_3_), which was presumed to be the aldehyde metabolite reduced from the carboxyl group and oxidized to a ketone at C3. The major fragment at *m*/*z* 133.0275 (C_8_H_5_O_2_) possessing the possible structure of 4-hydroxy-3-methylbenzaldehyde was formed by cleavage of the pyran ring. The fragment ions at *m*/*z* 255.1406, 199.0749, 171.0830, 135.0437 were all in good agreement with xiamenmycin A’s fragmentation pattern.

M3, eluted at 4.89 min, was characterized as the main metabolite, with the predominant quasi-molecular ion [M + H]^+^ at *m*/*z* 291.1596 (C_17_H_23_O_4_), which has the same molecular weight as xiamenmycin B. The structure was verified by authentic standards. It is interesting that there are at least three isomers observed in plasma, which are shown in Figure 4 (marked as M3′ and M3″) and which share the same fragmentation pattern as xiamenmycin B.

M4, eluted at 6.07 min, was characterized as the main metabolite, with the predominant quasi-molecular ion [M + H]^+^ at *m*/*z* 305.1753 (C_18_H_25_O_4_), which is 14 amu (−CH_2_) more than xiamenmycin B, suggesting a methyl ester of carboxylic acid in the side chain. The mass fragment of *m*/*z* 165.0544 is concordant with the fragmentation pattern of xiamenmycin B with an extra methyl group attached at the carboxyl group.

**Figure 5:**
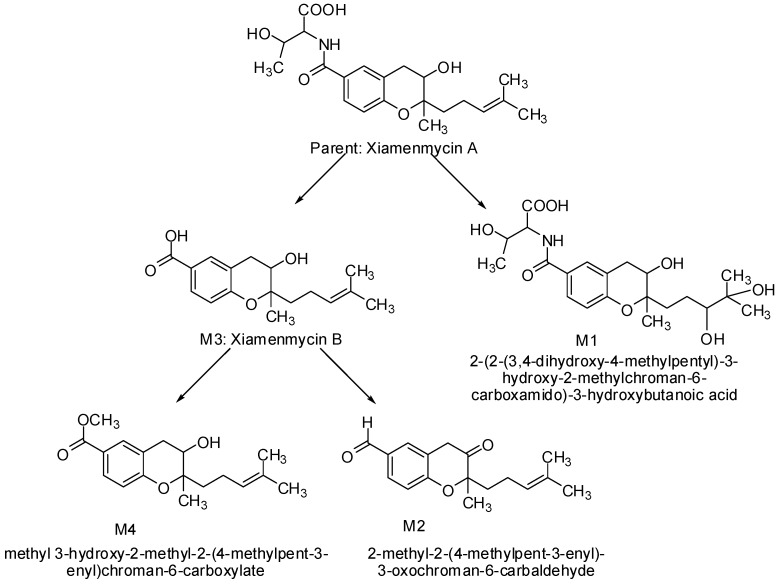
Proposed metabolic pathways of xiamenmycin A.

Four metabolites of xiamenmycin A were produced through hydrolysis and phase I (oxidation and reduction) and phase II (methyl conjugation) metabolism (Figure 5).

## 3. Discussion

Xiamenmycin A is an anti-fibrotic small molecule that targets the inflammatory and mechanical stress responses, the two pivotal pathological processes during excessive fibrogenesis [10]. Both *in vivo* and *in vitro* studies have found that the compound could suppress the proliferation, activation and contraction of fibroblasts, as well as inactivate FAK, p38 and Rho guanosine triphosphatase signaling [10]. This type of benzopyran compound was also found to inhibit the association of LFA-1 and ICAM-1 [16]. As a potential anti-fibrotic drug, the pharmacokinetic behavior and metabolites *in vivo* are crucial for pharmaceutical investigation.

We analyzed the fragment ions of xiamenmycins A–D to identify the MS/MS patterns. Xiamenmycins A and D possessed the same fragments at *m*/*z* 273.1487 and 255.1382 resulting from the loss of an amino acid moiety and a water molecule, respectively, as the characteristic mass fragment. The fragment ions for xiamenmycin B and C were formed by the cleavage of the pyran ring instead at *m*/*z* 151.0385 and 150.0547, respectively. The possible reason for different fragment patterns may be due to the two protonation modes, and the protonation occurring on the oxygen of pyran ring could trigger the cleavage of the C-O bond [17]. For xiamenmycin B, the [M + H]^+^ ions are mainly formed due to the attack of the proton of the oxygen atom in the pyran ring, which resulted in the easy cleavage of the ring [17]. On the other hand, xiamenmycins A and D possessed the amino acid moiety-containing amide group. The corresponding [M + H]^+^ ions of such a structure might be mainly generated by the attack of the proton of the nitrogen atom in the side chain instead of to the oxygen atom in the pyran ring. Thus, this may lead to the loss of the benzopyran skeleton as a complete fragment. It was reported that the degradation modes are always similar to the mass fragmentation patterns [13]. Thus, the fragmentation patterns are not only useful during structural determination, but also helpful during metabolite identification.

Time courses of the absorbed xiamenmycin in plasma after i.v. and i.p. administration were evaluated by relative quantification using LC-MS/MS. It was found that xiamenmycin A was absorbed into blood rapidly and eliminated quickly in plasma after comparison of the concentration curve post i.v. and i.p. administration. The rapid clearance of xiamenmycin may be improved by modification of the formulation. There was a minor difference in the clearance phase in the plasma concentration *vs.* time profiles for i.v. and i.p routes. The concentration of xiamenmycin A in blood reached a platform between 2 to 6 h, and clearance was reduced in the i.p. dosing when compared with the i.v. administration. Whether this phenomenon should be attributed to the initial storage in some organs and later release awaits further certification in future work. Our investigation of xiamenmycins is currently at the stage from discovery to clinical trials. Therefore, the investigations of pharmacokinetics and formulation selection are crucial for pharmaceutical development and will be carried out continuously.

Metabolite identification for the target compounds that undergo multiple and sequential metabolism remains a great challenge [13]. The metabolite analysis showed that xiamenmycin B is the principal form *in vivo* as the possible active form of xiamenmycin A after i.p. and i.v. administration. The structures of other metabolites were identified based on the characteristics of their MS and MS/MS data. In this study, the dynamic changes of xiamenmycin A in mouse plasma were investigated qualitatively, and the possible metabolites were identified by UPLC-QTOF-MS/MS. The metabolite analysis showed that xiamenmycin B is the principal form after i.p. and i.v. administration and is also the possible active form of xiamenmycin A. In our previous work, it was found that xiamenmycin A, C and D all possessed anti-fibrotic bioactivities, particularly xiamenmycin C, which is more active than the other two. Thus, the benzopyran skeleton is important for bioactivity. Xiamenmycin B might be the bioactive form of xiamenmycin A. It is interesting that there are possibly four isomers observed in plasma (shown in Figure 4, marked as M3′, M3″) that share the same fragmentation pattern as that of xiamenmycin B. M3', eluted at 3.87 min, showed the predominant quasi-molecular ion [M + H]^+^ at *m*/*z* 291.1593 (C_17_H_23_O_4_) and the fragment ions at *m*/*z* 273.1497, 203.0719, 189.0533 and 151.0382. M3″, eluted at 4.00 min, showed the predominant quasi-molecular ion [M + H]^+^ at *m*/*z* 291.1627 (C_17_H_23_O_4_) and the fragment ions at *m*/*z* 273.0871, 203.0712, 189.0538 and 151.0382. We proposed that they might be the stereo-isomers of xiamenmycin B or an isomer with a different hydroxylated position. Therefore, the metabolites of xiamenmycin A, namely xiamenmycin B, may be biotransformed through epimerization. However, because only one type of stereo-structure occurred naturally, the stereo-configurations of the metabolites need to be verified.

In summary, the present study provided important information concerning the metabolism of xiamenmycin A that is helpful for understanding the metabolic mechanism *in vivo*. As a promising candidate for treating excessive fibrotic disease, xiamenmycin A is a worthwhile compound to further investigate, including its pharmacokinetics and tissue distribution *in vivo*.

## 4. Experimental Section

### 4.1. General

Xiamenmycin A and B were isolated and purified in our laboratory [4]. The purity was 99%, as determined by HPLC. Acetonitrile and water were HPLC grade and purchased from Shanghai ANPEL Scientific Instrument Co. Ltd. (Shanghai, China). Formic acid was MS grade and was provided by Sigma-Aldrich (St. Louis, MO, USA). Verapamil was provided by Sigma-Aldrich (St. Louis, MO, USA). Organic solvents for LC-MS were of analytical grade and were purchased from Merck KGaA (Darmstadt, Germany).

### 4.2. Animals and Drug Administration

Xiamenmycin (5.95 mg) was dissolved in 30 μL of DMSO to prepare a stock solution. A 2-mg/mL xiamenmycin solution in 1% DMSO/water was prepared by diluting the stock solution with deionized water. According to our previous publication [10], xiamenmycin was administered to mice at a dose of 10 mg/kg by either i.v. or i.p. injection.

Female C57 mice (body weight, 15–18 g; 2007000565357) were obtained from SLAC Lab Animal Center of Shanghai (Shanghai, China). Food and water were provided *ad libitum*. The mice were fasted overnight before the day of the experiment, but had access to deionized water. Food was provided 4 h after administration. Eighteen mice were divided into six groups (A, B, C, D, E and F), each group containing three mice. The A–C groups were administered compounds intravenously, and the D–F groups were administered compounds intraperitoneally.

### 4.3. Sample Collection and Preparation

Blood samples (0.1–0.2 mL), obtained via the eyes, were collected in heparinized tubes. Samples from Groups A and D were collected at 0 min (0 h), 30 min (0.5 h), 4 h and 24 h. Samples from Groups B and E were collected at 5 min (0.08 h), 1 h and 6 h. Samples from Groups C and F were collected at 15 min (0.25 h), 2 h and 8 h. Next, all of the blood samples were centrifuged for 5 min at 8000 rpm, 4 °C, and the plasma was collected in a 96-well plate and stored at −20 °C. Thirty microliters of plasma were added to 90 μL of methanol containing 5 ng/mL verapamil, and the mixture was mixed by vortexing for 3 min followed by centrifugation at 15,000 rpm for 5 min. The supernatants were analyzed by LC-MS/MS.

Standard solutions for the quantitative determination of xiamenmycin were prepared by diluting the stock solution (2 mg/mL) to concentrations of 10, 20, 100, 500, 1000, 2000, 5000, 10,000, 20,000 and 50,000 ng/mL. The mixture of 30 μL of blank plasma and 3 μL of standard solution was mixed by vortexing for 3 min followed by centrifugation at 15,000 rpm for 5 min. The supernatants were analyzed by LC-MS/MS.

The remaining blood samples were extracted for metabolite analysis. Fifty microliters of plasma for each time point were used for metabolite extraction by adding 200 μL of a solution containing methanol and acetonitrile (5:3, V:V). After vortexing for 2 min, the mixture was kept at room temperature for 10 min and then centrifuged at 14,500× *g* for 20 min. The supernatant was collected and placed in the sampling vial for UPLC-QTOF-MS.

### 4.4. Instrumentation and Conditions

UPLC-QTOF-MS was performed using a Waters ACQUITY UPLC system equipped with a Micromass Q-TOF Premier mass spectrometer (Waters MS Technologies, Manchester, UK). Chromatographic separations were performed on a 2.1 × 100 mm (1.7 μm) ACQUITY BEH C18 chromatography column. The column temperature was set at 45 °C, and the gradient eluting program was started from 5% B, changed to 30% B within 2 min, to 60% B within 2.5 min, then changed to 100% B in another 7.5 min and, at last, held at 100% B for 2 min (Solvent A: aqueous solution of 0.1% formic acid; Solvent B: ACN of 0.1% formic acid). The total flow rate was 0.40 mL/min. The eluate was directed to the mass spectrometer without splitting. Mass analysis was performed using a Q-TOF mass spectrometer equipped with an ESI source operating in the positive and negative ion modes. The desolvation and cone gas rate were set at 600 L/h at a temperature of 350 °C and 50 L/h, respectively. The source temperature was set at 110 °C. The capillary and cone voltages were set at 3000 and 35 V in the positive mode and at 2800 V and 25 V in the negative mode, respectively. The collision energy for the MS scan was 6 eV; for the MS/MS scan, the collision energy ramped up from 15 eV to 30 eV. Data were acquired in the centroid mode from the mass-to-charge ratio (*m*/*z*) 100 to 1000 at a scan time of 0.25 s with a lock spray frequency of 15 s. A lock mass of leucine encephalin ([M + H]^+^ = 556.2771, [M − H]^−^ = 554.2615) at a concentration of 200 ng/mL was used via a lock spray interface at a flow rate of 0.02 mL/min to ensure mass accuracy during the MS analysis.

Mass spectrum analysis was carried out using Metabolynx™ and Masslynx 4.1 software (Waters MS Technologies, Manchester, UK).

LC-MS/MS was performed on an Agilent HPLC 1290 system and 6460 series triple quadrupole mass spectrometer (Agilent Technologies, Waldbronn, Germany). Chromatographic separations were performed on a 2.1 × 100 mm (3.5 μm) Zorbax SB-C8 Narrow Bore EE chromatography column. The column temperature was set at 30 °C at a flow rate of 0.30 mL/min. The gradient eluting program was started from 45% B to 85% B within 2 min and held at 85% B for 0.8 min, where Solvent A was an aqueous solution of 0.1% formic acid and 5 mM ammonium formate and Solvent B was methanol. The injection volume was 5 μL. The optimized ESI instrument parameters for the mass spectrometry measurement using Agilent Mass Hunter WorkStation software B.04.01 were as follows: multiple reaction monitoring (MRM) mode; drying gas flow rate: 10 L·min^−1^; drying gas temperature: 350 °C; nebulizer gas pressure: 30 psi; capillary voltage: 3500 V. The transitions (precursor/product ion pair) were 392.2/273.2 for xiamenmycin A with a collision energy of 8 eV and 455.2/165.1 for verapamil (internal standard) with a collision energy of 25 eV.

## 5. Conclusions

We analyzed the fragmentation ions of xiamenmycins A–D to identify the MS/MS patterns. Xiamenmycins A and D possessed the same fragments resulting from the loss of the amino acid moiety and hydroxyl group, respectively, as the characteristic mass fragment. Xiamenmycin B and C shared the fragments formed by cleavage of the pyran ring. The time course of the absorbed xiamenmycin in plasma was evaluated semi-quantitatively for future pharmacokinetic experiments by the LC-MS/MS method. It was found that xiamenmycin A was absorbed into blood rapidly and was eliminated quickly in plasma. A total of four metabolites and the parent compound, xiamenmycin A, were identified in plasma qualitatively. The metabolite analysis showed that xiamenmycin B is the principal form *in vivo* after i.p. and i.v. administration, confirmed by matching the retention time and mass fragments with a standard sample. The structures of other metabolites were identified by the analysis of the mass fragment patterns. The results indicate that hydrolysis, oxidation, reduction and methyl conjugation were the major metabolic pathways. In summary, the dynamic changes of xiamenmycin A concentration in mouse plasma were investigated, and the possible metabolites were identified by UPLC-QTOF-MS. The newly identified metabolites are useful for understanding the metabolism of xiamenmycin A *in vivo*, aiming to develop anti-fibrotic drug candidates for the therapeutic treatment of excessive fibrotic diseases.

## Supplementary Files

Supplementary File 1
