# Supplementary material for: In Vivo Metabolism Study of Xiamenmycin A in Mouse Plasma by UPLC-QTOF-MS and LC-MS/MS
Source: Mar Drugs. 2015 Jan 28;13(2):727–40. doi: 10.3390/md13020727 (PMC4344598; doi:10.3390/md13020727)
Supplement: Supplementary File 1 [file marinedrugs-13-00727-s001.pdf]

Supplementary Information

Figure S1. Linear standard curve of xiamenmycin A S2
Table S1. Time-course of xiamenmycin A after i.v. administration S2
Table S2. Time-course of xiamenmycin A after i.p. administration S2
Figure S2. Secondary mass spectrum of xiamenmycin A S3
Figure S3. Secondary mass spectrum of xiamenmycin B S3
Figure S4. Secondary mass spectrum of xiamenmycin C S4
Figure S5. Secondary mass spectrum of xiamenmycin D S4
Figure S6. Secondary mass spectrum of M1 .... S5
Figure S7. Secondary mass spectrum of M2 .... S5
Figure S8. Secondary mass spectrum of M3 .... S6
Figure S9. Secondary mass spectrum of M4 .... S6
Figure S10. Secondary mass spectrum of parent compound in plasma S7

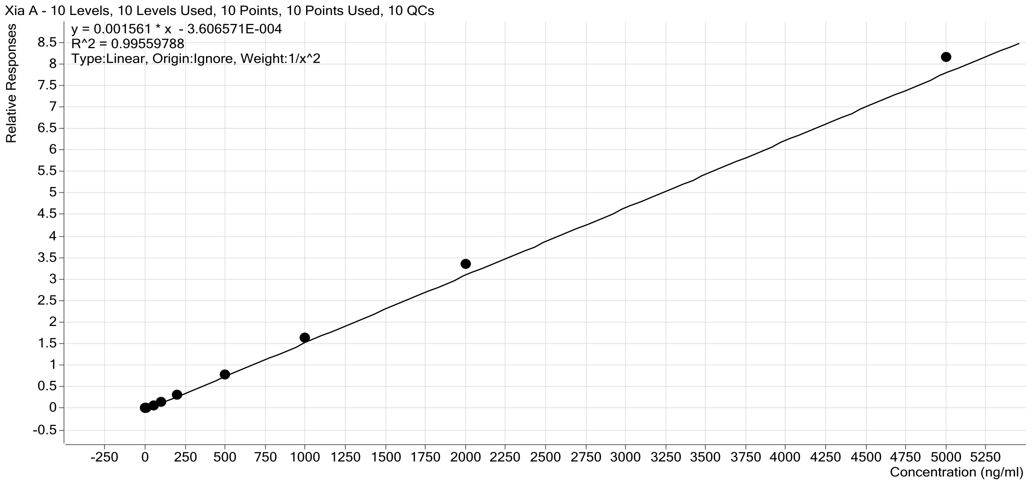

Figure S1. Linear standard curve of xiamenmycin A.

Table S1. Time-course of xiamenmycin A after i.v. administration.

**Time/h**	**♀**			**Mean**	**SD**
	**1**	**2**	**3**		
0.00	0.00	0.00	0.00	0.00	0.00
0.08	10,666.25	12,977.75	13,514.86	12,386.28	1513.61
0.25	4277.27	2014.05	2627.25	2972.86	1170.53
0.50	373.20	1703.56	642.62	906.46	703.33
1.00	121.90	96.35	99.86	106.03	13.85
2.00	48.97	28.09	26.38	34.48	12.58
4.00	13.90	13.25	17.70	14.95	2.40
6.00	10.15	14.92	5.66	10.24	4.63
8.00	3.21	11.10	7.28	7.20	3.95
24.00	BQL	1.04	1.17	1.11	0.09

BQL: Below the limitation.

Table S2. Time-course of xiamenmycin A after i.p. administration.

**Time/h**	**♀**			**Mean**	**SD**
	**1**	**2**	**3**		
0.00	0.00	0.00	0.00	0.00	0.00
0.08	8975.60	10,446.04	8351.13	9257.59	1075.55
0.25	3921.73	4470.94	4720.15	4370.94	408.50
0.50	1194.24	1116.63	1944.08	1418.32	456.97
1.00	131.05	88.93	85.75	101.91	25.28
2.00	18.45	27.26	14.09	19.93	6.71
4.00	20.31	19.46	17.93	19.23	1.21
6.00	12.23	22.17	17.18	17.19	4.97
8.00	4.15	4.78	5.59	4.84	0.72
24.00	1.22	1.53	1.58	1.44	0.20

BQL: Below the limitation.

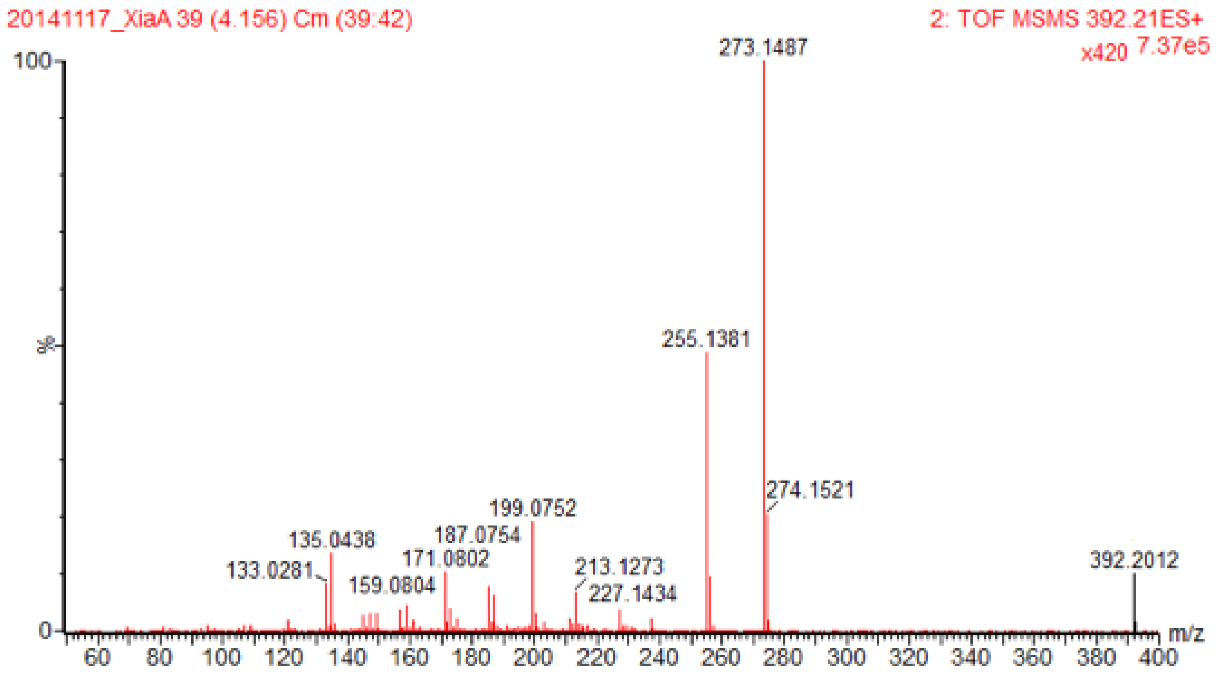

Figure S2. Secondary mass spectrum of xiamenmycin A.

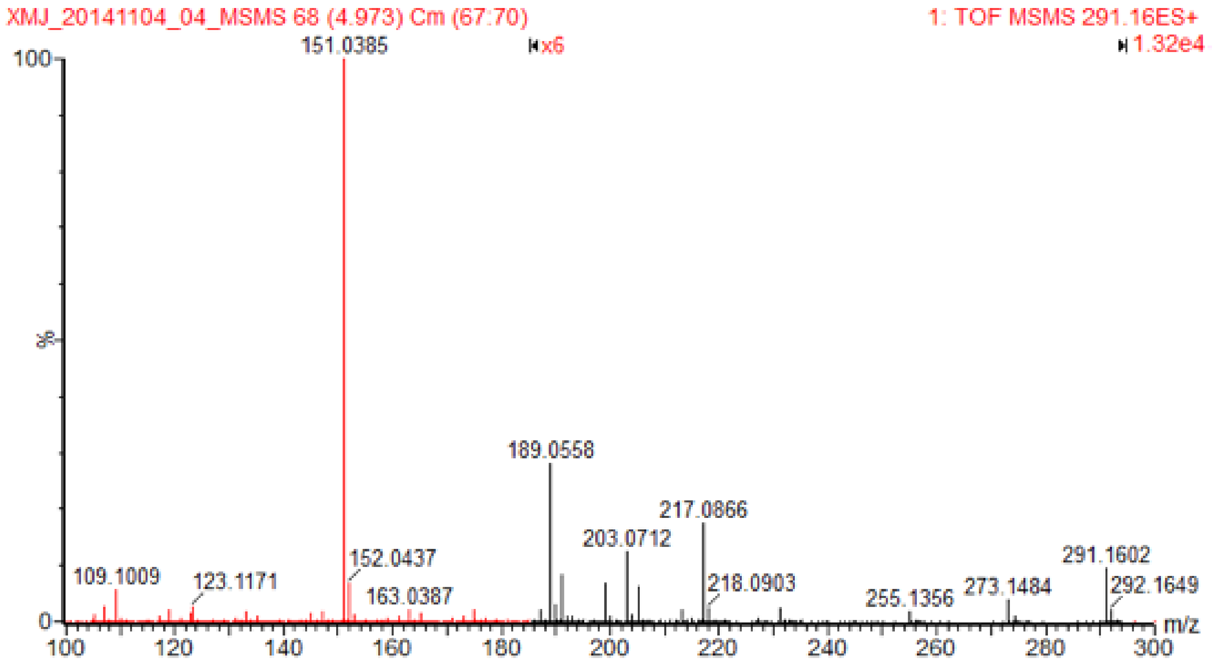

Figure S3. Secondary mass spectrum of xiamenmycin B.

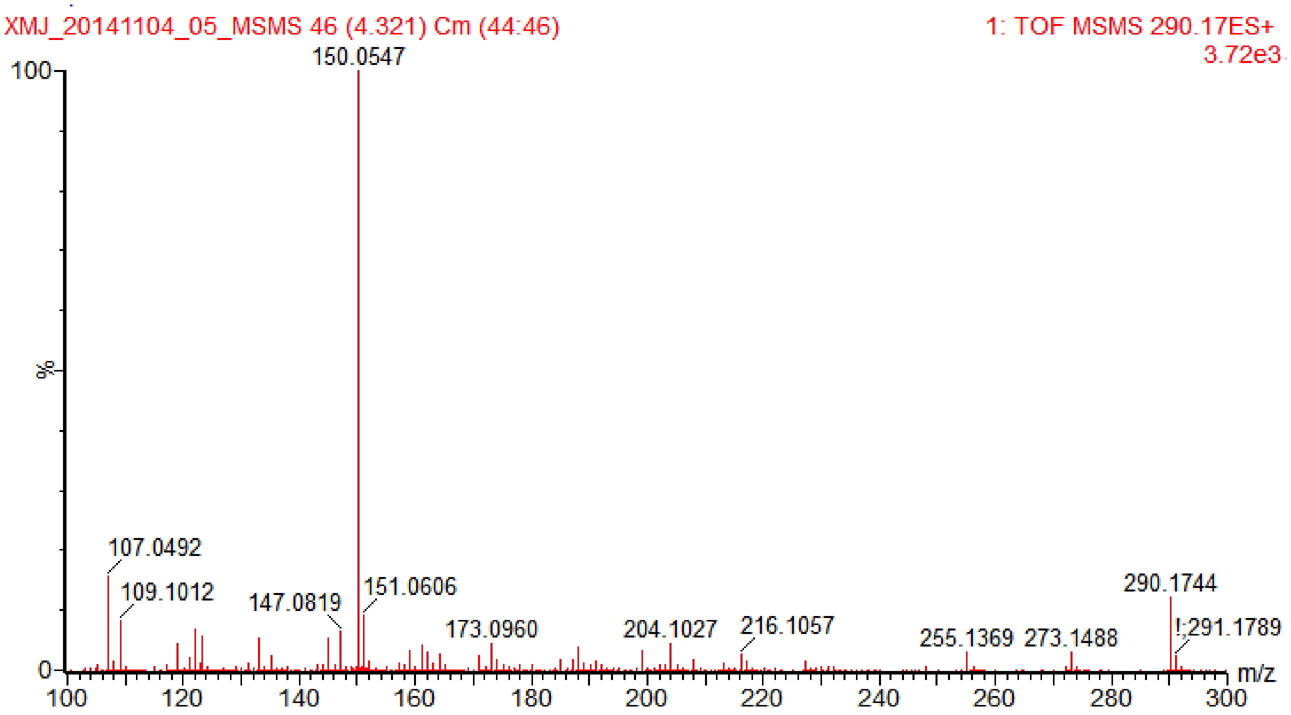

Figure S4. Secondary mass spectrum of xiamenmycin C.

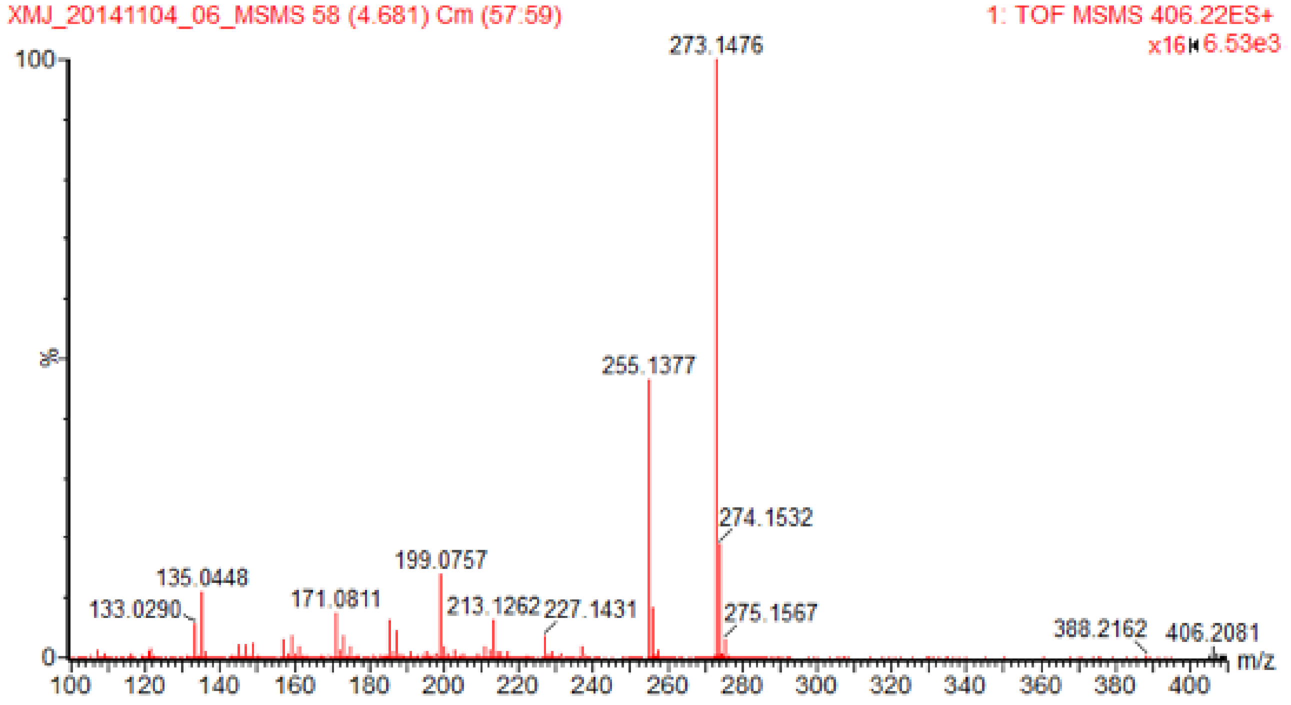

Figure S5. Secondary mass spectrum of xiamenmycin D.

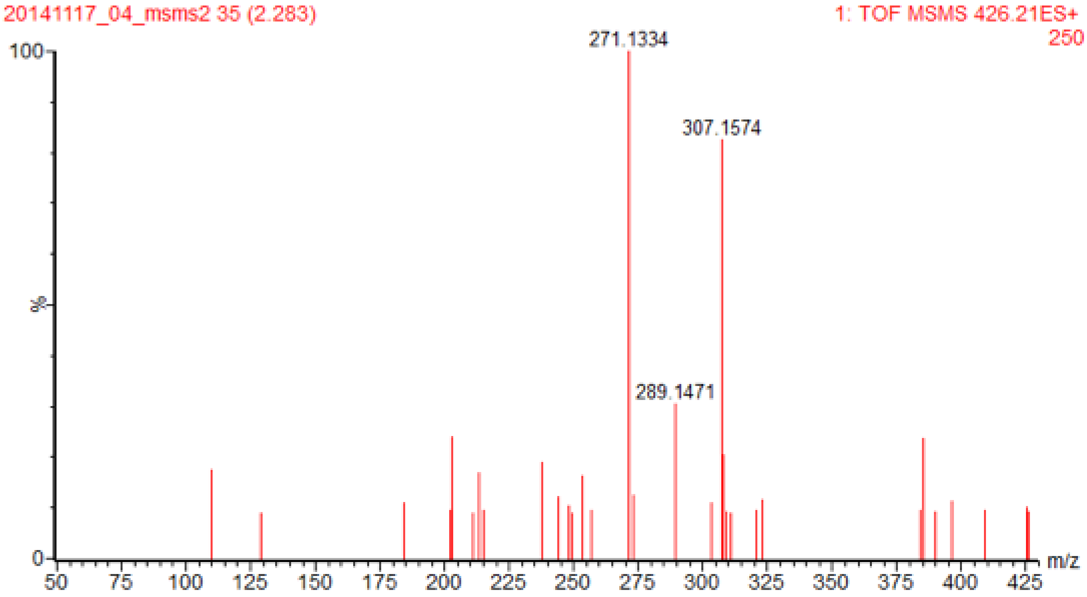

Figure S6. Secondary mass spectrum of M1.

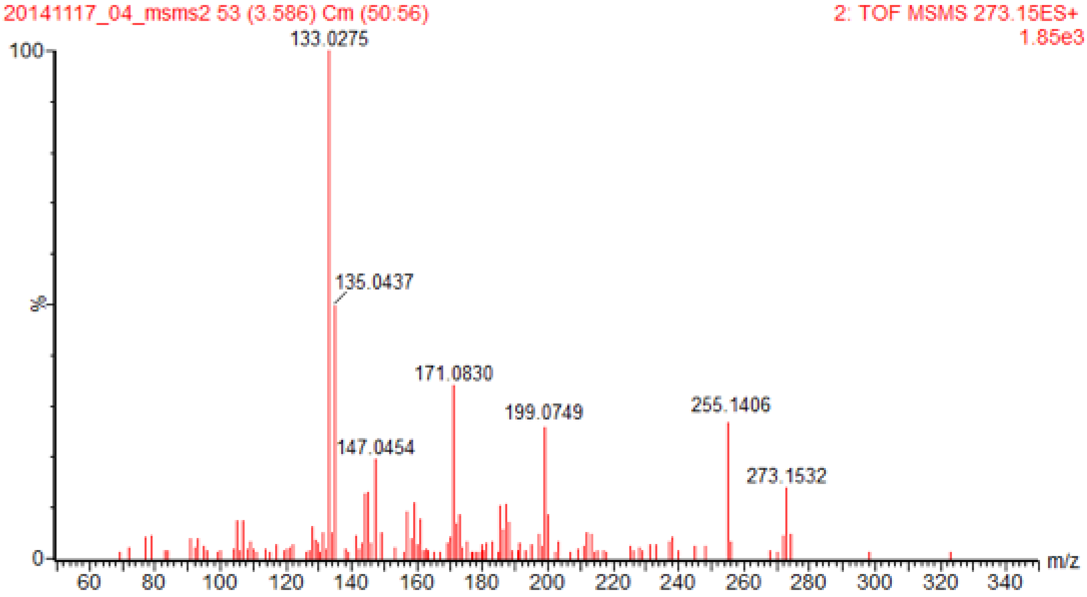

Figure S7. Secondary mass spectrum of M2.

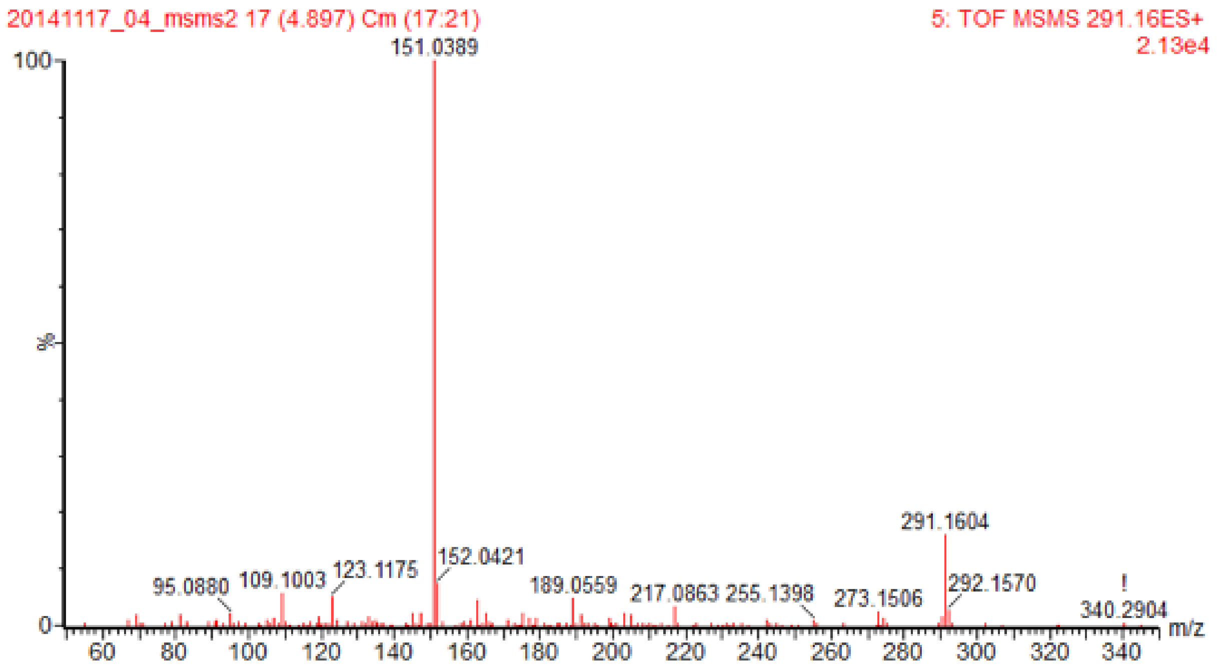

Figure S8. Secondary mass spectrum of M3.

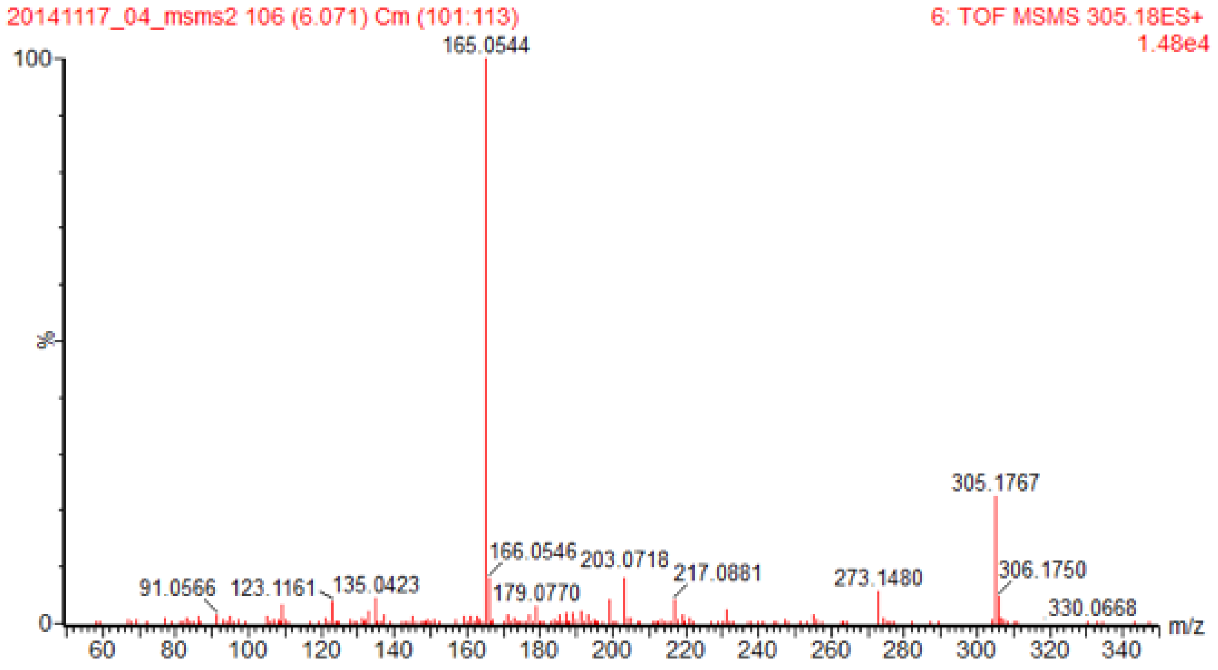

Figure S9. Secondary mass spectrum of M4.

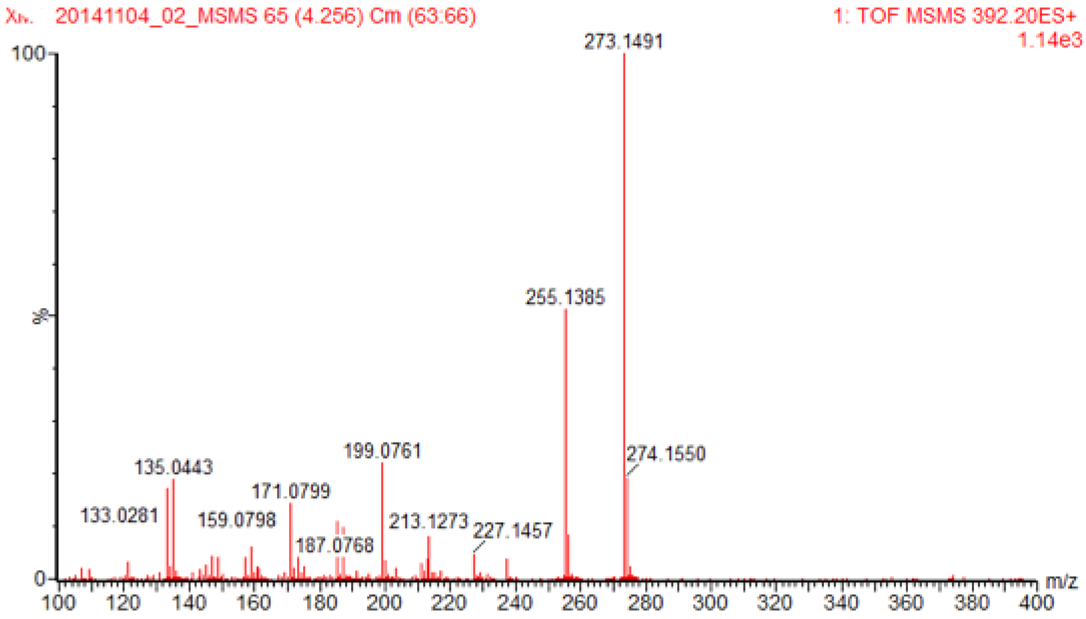

Figure S10. Secondary mass spectrum of parent compound in plasma.

© 2015 by the authors; licensee MDPI, Basel, Switzerland. This article is an open access article distributed under the terms and conditions of the Creative Commons Attribution license (http://creativecommons.org/licenses/by/4.0/).
